# Supplementary material for: Subversion of the Heme Oxygenase-1 Antiviral Activity by Zika Virus
Source: Viruses. 2018 Dec 20;11(1):2. doi: 10.3390/v11010002 (PMC6356520; doi:10.3390/v11010002)
Supplement: Supplementary file 1 [file viruses-11-00002-s001.pdf]

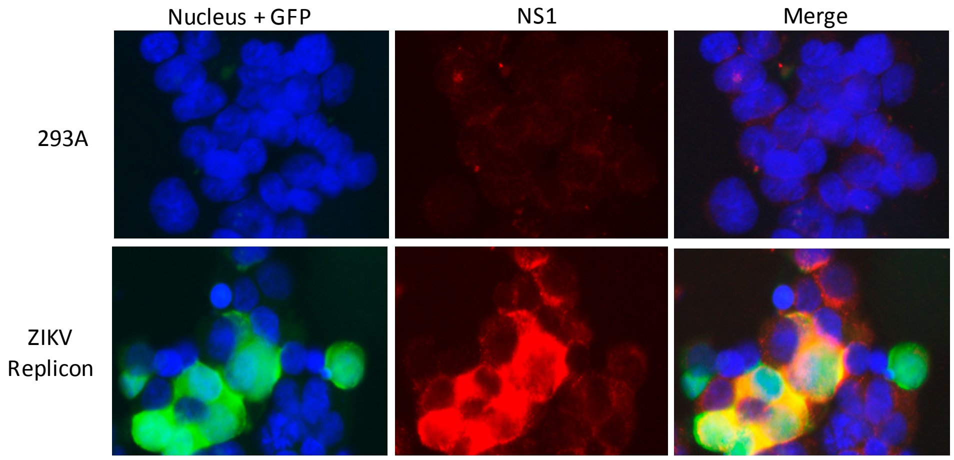

S1. Supplemental data

NS1 expression in ZIKV replicon cells. Fluorescence microscopy images of ZIKV replicon cells (GFP positive) after immunostaining of NS1 (red) with antibody against NS1 (abcam).

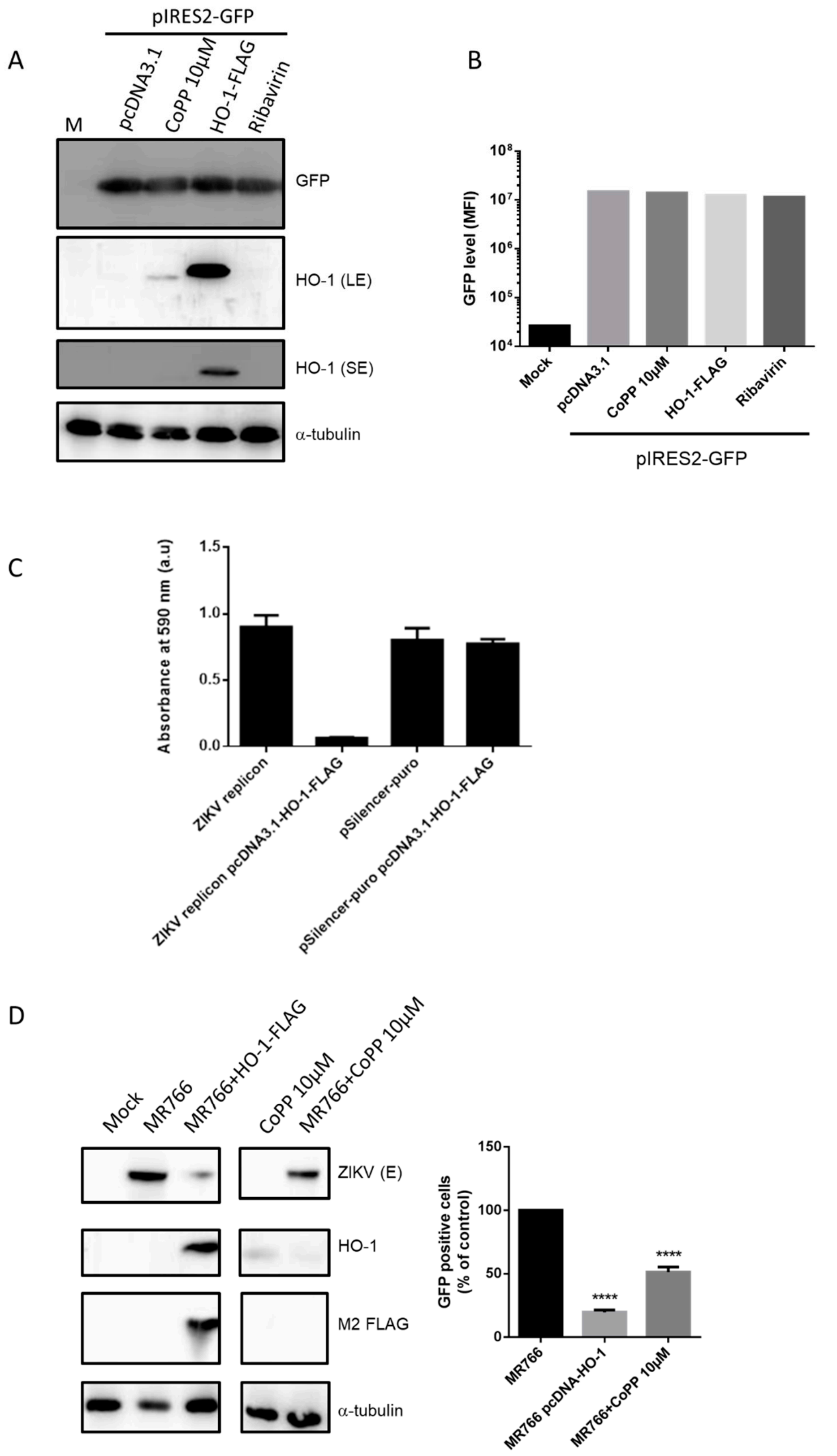

S2. Supplemental data

A. and B. Overexpression or induction by CoPP of HO-1 do not interfere with CMV driven GFP expression. HEK-293T were transfected with pIRES2-GFP or mock transfected. 24h after cells were transfected with pcDNA3.1-HO-1-FLAG or treated with CoPP at 10 μM or Ribavirin (164μM) for 16h. After treatment cells were harvest for western blot (A) or cytometer analysis (B). (LE: Long exposure, SE: short exposure).

C. Overexpression of HO-1 in HEK-293A cells does not induce mortality. HEK-293A cells were transfected with pSilencer-Puro plasmid (Ambion) with lipofectamine after 5 days of growth with 1μg.mL^{-1} of puromycin and cells were transfected with pcDNA3.1 or pcDNA3.1-HO-1-FLAG, after 24h, cells were treated for 7 days of with G418 and puromycin and stain with crytal violet.

D. Overexpression or induction by CoPP of HO-1 are able to downregulate MR766-GFP ZIKA virus replication. 293T were transfected with pcDNA3.1-HO-1-FLAG followed by infection with MR766-GFP ZIKA. For induction with CoPP, 2h post-infection, CoPP were added at 10 μM for 16h.and infected or treated with CoPP at 10 μM. After treatment cells were harvest for western blot or cytometer analysis.

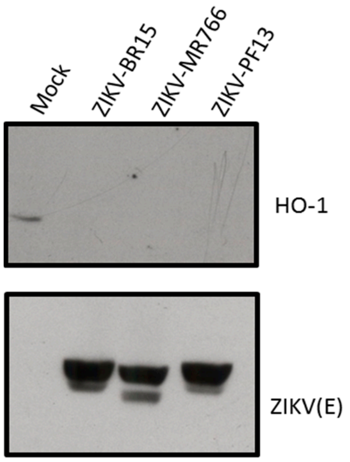

S3. Supplemental data

Different lineages of ZIKV down-regulate HO-1 expression. Western blot analysis of HO-1 expression during ZIKV infection with different lineages in Vero cells after 48h at MOI of 1. Viruses have been described previously [13]. Expression of E-ZIKV protein was followed using the pan flavivirus antibody 4G2.

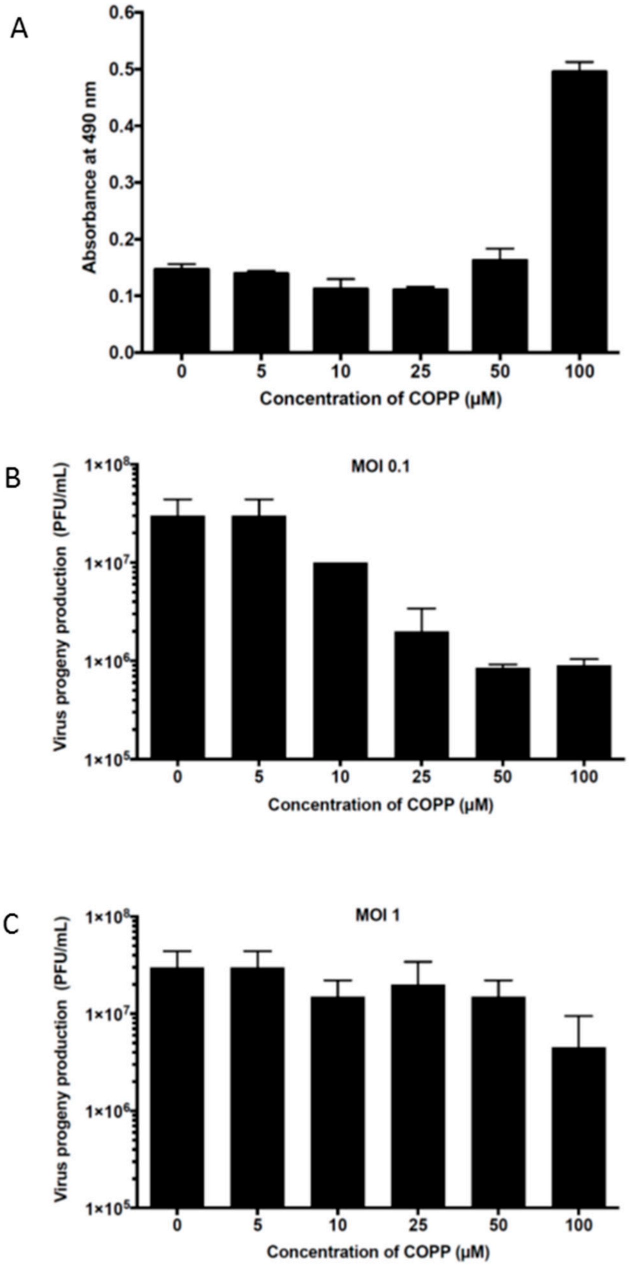

S4. Supplemental data

Induction of HO-1 with CoPP decreases the viral progeny production of ZIKV-PF13 only at lower MOI. (A) Vero cells were treated with different concentration of CoPP during 48 h. LDH release was quantified at 48 h post-treatment. Vero cells were infected with ZIKV-PF13 at MOI of 0.1 **(B)** and MOI of 1 **(C)**. 2 h post infection CoPP were added at different concentrations. Viral progeny production was determined after 24 h of infection.
